# The curves of elliptic nodes on K3 surfaces

**DOI:** 10.1007/s13366-026-00829-x

**Published:** 2026-03-11

**Authors:** Jiexiang Huang

**Affiliations:** Mathematical Institute, Universität Bonn, 53113 Bonn, Germany

**Keywords:** Chow groups, Curves, K3 surfaces

## Abstract

Let (*X*, *L*) be a polarized K3 surface of genus *g* and $C_{en} \subset X$ be the curve of singular points of nodal elliptic curves in |*L*|. When (*X*, *L*) is generic of genus two, Huybrechts proved that the curve $C_{en}$ is a constant cycle curve and conjectured that this remains true for higher genus cases. In this note, we show that the conjecture holds true for polarized K3 surfaces (*X*, *L*) lying in a locus of codimension one in the moduli space of polarized K3 surfaces of genus *g* for every g>2

## Introduction

Constant cycle curves on complex projective K3 surfaces are curves whose points represent the same class in $CH _0(X)$. Rational curves on K3 surfaces are the simplest examples and those of higher genus were systematically studied in Huybrechts (2014). Among them, a particularly interesting example arises from curves in the fixed locus of a non-symplectic automorphism of a K3 surface (see Huybrechts 2014, Prop. 7.1).

In this note, we study further concrete examples of constant cycle curves on K3 surfaces. A natural candidate for a constant cycle curve on a generic polarized K3 surface (*X*, *L*) of genus *g*, suggested by Huybrechts, is constructed as follows. Recall from Mori and Mukai (1983) that for a generic K3 surface there exists a one-dimensional family of nodal elliptic curves in |*L*|. Their singularities sweep out a curve $C_{en}$ (up to taking the closure) on *X* and we call it the *curve of elliptic nodes* of (*X*, *L*).

When g=2, Huybrechts observed that the curve $C_{en}$ coincides with the fixed locus of the natural involution on *X*, thus is a constant cycle curve. However, a similar geometric interpretation for $C_{en}$ no longer exists when the genus of (*X*, *L*) is higher. We wonder whether the curve of elliptic nodes $C_{en}$ in the cases g⩾3 nevertheless still offers new examples of constant cycle curves. This led Huybrechts to conjecture that

### Conjecture 0.1

(Huybrechts) Let (*X*, *L*) be a polarized K3 surface of genus g⩾3. Then the curve $C_{en}$ of elliptic nodes of (*X*, *L*), if nonempty, is a constant cycle curve.

For non-generic polarized K3 surfaces, the nonemptiness of $C_{en}$ is only known in certain special cases. For details we refer to Sect. 2.2.

In this paper, we give the following partial answer to Conjecture 0.1 (see Sect. 2.3 for the proof).

### Theorem 0.2

Let $\mathcal {F}_g$ be the moduli space of polarized K3 surfaces of genus *g*. For each g>2, there exists a locus of codimension one in $\mathcal {F}_g$ for which Conjecture 0.1 holds.

This result is obtained by studying the curves of elliptic nodes on hyperelliptic K3 surfaces. According to Reid (1976), these K3 surfaces can be realized as double covers of Hirzebruch surfaces or $\mathbb {P}^2$. Those that are double covers of $\mathbb {F}_0$ (resp. $\mathbb {F}_1$), endowed with appropriate polarizations, form an 18-dimensional locus in the moduli space $\mathcal {F}_g$ of polarized K3 surfaces of any odd genus g⩾3 (resp. even genus g⩾4); see Sect. 2.1 for a brief summary.

In Sect. 2.2, we show the nonemptiness of $C_{en}$ for a generic hyperelliptic K3 surface in $\mathcal {F}_g$. For such a polarized K3 surface, similar to the genus two case, we prove in Sect. 2.3 that the curve $C_{en}$ can be identified with the fixed locus of the hyperelliptic involution. Therefore, $C_{en}$ is a constant cycle curve by Huybrechts (2014, Prop. 7.1), and Theorem 0.2 follows immediately. We also provide an alternative proof in Remark 2.10 that does not rely on the fixed curve property of $C_{en}$.

For generic polarized K3 surfaces of genus g>2, Conjecture 0.1 remains open. The major difficulty is a lack of an alternative geometric interpretation for the distinguished curve $C_{en}$, even if the projective model of *X* is known, e.g. when (*X*, *L*) is generic of small genus. Such an interpretation would enable us to compare the cycle classes of closed points on $C_{en}$ in $CH _{0}(X)$.

In Sect. 3, we study two conditions on nodal elliptic curves in |*L*| that imply $C_{\text {en}}$ is a constant cycle curve. The first is applicable to the study of Conjecture 0.1 for generic polarized K3 surfaces, while the second is merely a sufficient condition that holds precisely for hyperelliptic K3 surfaces.

*Notations and conventions.* We always work over the base field $\mathbb {C}$. A nodal elliptic curve in our convention is an integral nodal projective curve of geometric genus one. The coarse moduli space of polarized K3 surfaces of genus *g* is denoted by $\mathcal {F}_g$. We say a property holds for a *generic* polarized K3 surface of genus *g* if it holds for points (*X*, *L*) in some non-empty Zariski open subset of $\mathcal {F}_g$.

## Preparations

In this section, we motivate the study of Conjecture 0.1 and give the formal definition of the curve of elliptic nodes $C_{en}$ of a generic polarized K3 surface (*X*, *L*) of genus *g*.

### Constant cycle curves

Let us first review some basic facts about constant cycle curves on K3 surfaces following Huybrechts (2014). Throughout this part, *X* denotes a projective K3 surface over $\mathbb {C}$.

#### Definition 1.1

A curve C⊂X is a constant cycle curve^1^ if [x]=[y] in $CH _0(X)$ for any two closed points x,y∈C.

Recall from Beauville and Voisin (2004) that the group $CH _0(X)$ contains a canonical class $c_X$, called the *Beauville–Voisin class*. This class can be realized by any point lying on a rational curve on *X*, and has the key property that the intersection product of any two divisors on *X* is a multiple of $c_X$. From Voisin (2015, Lem. 2.2) it follows that every point on a constant cycle curve represents the Beauville–Voisin class $c_X \in CH _0(X)$.

The following result (Huybrechts 2014, Prop. 7.1) provides a method to produce constant cycle curves on K3 surfaces that admit non-symplectic automorphisms.

#### Proposition 1.2

Let *f* be a non-symplectic automorphism of finite order *n* of a K3 surface *X*. Then any curve C⊂X contained in the fixed locus of *f* is a constant cycle curve.

Note that by Mumford (1969) and the verified case of Bloch’s conjecture in Bloch et al. (1976), an automorphism *f* is non-symplectic if and only if the quotient surface $\bar{X} :=X/\langle f \rangle$ satisfies $CH _0(\bar{X}) \simeq \mathbb {Z}$. In particular, for a generic polarized K3 surface of genus two, i.e. a double cover $\pi :X \rightarrow \mathbb {P}^2$ ramified over a smooth sextic $B \in |\mathcal {O}_{\mathbb {P}^2}(6)|$, the fixed curve $D :=\pi ^{-1}(B) \subset X$ of the natural involution of *X* is a constant cycle curve (Huybrechts 2014, Sec. 7.2).

### The curve of elliptic nodes of (*X*, *L*)

We are interested in constructing constant cycle curves on a generic polarized K3 surface $(X,L) \in \mathcal {F}_{g}$ of genus g⩾3. Such a K3 surface does not admit a non-symplectic automorphism, hence Proposition 1.2 cannot be applied to them and one needs new constructions.

For a generic polarized K3 surface (*X*, *L*) of genus two, Huybrechts observed that the ramification curve *D* of the double cover $\pi :X \rightarrow \mathbb {P}^2$ is swept out by the nodes of nodal elliptic curves in the linear system $|L| = |\pi ^{*}\mathcal {O}_{\mathbb {P}}(1)|$. Indeed, each nodal elliptic curve in |*L*| is the pullback of a simple tangent line to the branch curve $B \in |\mathcal {O}_{\mathbb {P}^2}(6)|$, with its node lying over the point of tangency. This alternative geometric description of the curve *D* suggests a natural candidate for a constant cycle curve on any polarized K3 surface of higher genus, as follows.

Let (*X*, *L*) be a polarized K3 surface of genus *g*. A nodal elliptic curve in |*L*| possesses exactly (g-1) nodal points. The locus of such curves in |*L*|, known as the *Severi variety* $V_{L,g-1} \subset |L|$, is smooth of dimension one once it is nonempty (Chiantini and Sernesi 1996, Ex. 1.3). We consider the following curve on the K3 surface *X* swept out by singularities of nodal elliptic curves in |*L*|.

#### Definition 1.3

Let (*X*, *L*) be a polarized K3 surface of genus *g*. *The curve of elliptic nodes* $C_{en}$ of (*X*, *L*) is defined as the union of one-dimensional components of the closure of the following set

{$P \in X \mid P$ is the nodal point of some nodal elliptic curve E∈|L|}

with the reduced scheme structure (we allow $C_{en}$ to be empty if there exist no nodal elliptic curves in |*L*|).

#### Remark 1.4

The closure of the above set of singularities has dimension one once the Severi variety $V_{L,g-1}$ is nonempty, for instance when (*X*, *L*) is generic in the moduli space (Mori and Mukai 1983). However, we do not know if the closure is always irreducible or even of pure dimension one. Therefore, we discard discrete points that possibly exist in the definition of $C_{en}$. This is unnecessary when (*X*, *L*) is generic of genus three: The curve of elliptic nodes $C_{en}$ of a smooth quartic, studied in Witaszek (2014) under the name “double-cover curve", is known to be irreducible of degree 320 and genus 1281, with only nodal and cusp singularities.

From the toy example of ramification curves of double planes we obtain

#### Corollary 1.5

The curve of elliptic nodes $C_{en}$ of a generic polarized K3 surface (*X*, *L*) of genus two is a constant cycle curve.

## The conjecture for hyperelliptic K3 surfaces

The aim of this section is to prove our main result Theorem 0.2.

### Review on hyperelliptic K3 surfaces

We collect here some facts about hyperelliptic K3 surfaces from Saint-Donat (1974) and Reid (1976) for later use.

#### Theorem 2.1

(Saint-Donat 1974, Sec. 4, Cor. 5.8) Let *X* be a K3 surface and *L* be a line bundle on *X* with $(L)^2 = 2g-2 > 0$. Suppose the linear system |*L*| has no fixed components. Then |*L*| has no base points and induces a morphism $\phi _L :X \rightarrow \mathbb {P}^{g}$, which is

(i) either generically 2 : 1 onto its image; or
(ii) birational onto its image.

The first case occurs if and only if any smooth irreducible member of |*L*| is a hyperelliptic curve.

This yields the following definition of hyperelliptic K3 surfaces.

#### Definition 2.2

A base point free complete linear system |*L*| on a K3 surface *X* is *hyperelliptic* if the induced morphism $\phi _{L}$ is generically 2 : 1 onto its image. We say a K3 surface *X* is *hyperelliptic* if it admits a hyperelliptic linear system |*L*|, and we will call the pair (*X*, *L*) a *hyperelliptic* K3 surface when the hyperelliptic linear system |*L*| is assigned.

Hyperelliptic K3 surfaces are natural generalizations of double planes, i.e. K3 surfaces that can be realized as double covers of $\mathbb {P}^2$. By Reid (1976), a hyperelliptic K3 surface *X* is always a double cover of $\mathbb {P}^2$ or of the *n*-th Hirzebruch surface $\mathbb {F}_n = \mathbb {P}(\mathcal {O}_{\mathbb {P}^1} \oplus \mathcal {O}_{\mathbb {P}^1}(-n))$, where $0 \leqslant n \leqslant 4$. Conversely, one can construct hyperelliptic K3 surfaces as double covers of $\mathbb {F}_n$ as follows. Let $p :\mathbb {F}_n \rightarrow \mathbb {P}^1$ be the natural projection. Recall that $Pic (\mathbb {F}_n) \simeq \mathbb {Z}e \oplus \mathbb {Z}f$, where *e* is the unique section class representing $\mathcal {O}_{p}(1)$ and *f* is the fibre class. The two generators satisfy the numerical relations

1 $$\begin{aligned} e^2 = -n, \ \ ef = 1, \ \ f^2 = 0. \end{aligned}$$

In addition, for an integer *r*, the line bundle $\mathcal {O}(e+rf)$ on $\mathbb {F}_n$ is ample if and only if r>n, see e.g. (Hartshorne 1977, Ch.V, Cor 2.18). Then we have the following construction:

#### Proposition 2.3

(Reid 1976) Fix an integer $0 \leqslant n \leqslant 4$. Let $B \in |-2K_{\mathbb {F}_n}|$ be a smooth curve and $\pi :X \rightarrow \mathbb {F}_n$ be the double cover ramified over *B*. Consider the line bundle $L_{r} :=\pi ^{*}\mathcal {O}(e+rf)$ on *X* for an integer r>n. Then $(X,L_r)$ is a polarized K3 surface of genus g=2r+1-n. In addition, $(X,L_r)$ is hyperelliptic.

The cases n=0 and n=1 are of particular importance to us. Note that $\mathbb {F}_0 \simeq \mathbb {P}^1 \times \mathbb {P}^1$ and $\mathbb {F}_1 \simeq Bl _P(\mathbb {P}^2)$, the blow-up of $\mathbb {P}^2$ at one point *P*. The dimension of the corresponding hyperelliptic locus in $\mathcal {F}_g$ is computed as follows.

#### Proposition 2.4

(Reid 1976) Let n=0 or 1 and r>n. Write $\mathcal {F}_{n,r}$ for the locus of pairs $(X,L_r)$ in the moduli space $\mathcal {F}_{g}$ of polarized K3 surfaces of genus g=2r+1-n. Then one has

$$ \dim \mathcal {F}_{n,r} = \dim |-2K_{\mathbb {F}_{n}}| - \dim Aut (\mathbb {F}_{n}) = 18. $$

In other words, the family of hyperelliptic K3 surfaces that are double covers of $\mathbb {F}_0$ (resp. $\mathbb {F}_1$), equipped with the polarization $|L_r|$, forms a divisor in the moduli space $\mathcal {F}_g$ of polarized K3 surfaces of genus g=2r+1⩾3 (resp. g=2r⩾4). Varying the integer r>n thereby realizes all genera g⩾3.

#### Remark 2.5

Hyperelliptic K3 surfaces that are double covers of $\mathbb {F}_1$ arise from degenerations of double planes, since the isomorphism $\mathbb {F}_1 \simeq Bl _{P}(\mathbb {P}^2)$ identifies a generic smooth curve $B \in |-2K_{\mathbb {F}_1}| = |\mathcal {O}(6(f+e) - 2e)|$ with the strict transform of a plane sextic acquiring exactly one node. In contrast, hyperelliptic K3 surfaces that are double covers of $\mathbb {F}_0$ cannot be obtained similarly, since there is no regular map from $\mathbb {F}_0 \simeq \mathbb {P}^1 \times \mathbb {P}^1$ to $\mathbb {P}^2$. By blowing up one point $P \in \mathbb {F}_0$ and then contracting the strict transforms of the two rulings through *P*, we obtain a regular morphism $Bl _P(\mathbb {F}_0) \rightarrow \mathbb {P}^2$. One then finds that the strict transform $\tilde{B} \subset Bl _P(\mathbb {F}_0)$ of a smooth curve $B \in |-2K_{\mathbb {F}_0}|$ does not map to a plane sextic under this morphism, regardless of whether *B* contains the blow-up point *P*.

### Nonemptiness of the curve $C_{en}$

For a generic polarized K3 surface (*X*, *L*), the linear system |*L*| contains a one-dimensional family of nodal elliptic curves (Mori and Mukai 1983), thus the curve of elliptic nodes $C_{en}$ is nonempty. Since hyperelliptic K3 surfaces form only a divisor in $\mathcal {F}_g$, the curve $C_{en}$ could a priori be empty for them. As we explain below it follows from recent work by Chen et al. (2022) that for a generic hyperelliptic K3 surface of genus *g* the curve $C_{en}$ is not empty.

First, we fix some relevant conventions on lattice polarized K3 surfaces.

#### Definition 2.6

Let Λ be a lattice, i.e. a free $\mathbb {Z}$-module of finite rank equipped with a symmetric bilinear form. An *ample* Λ*-polarized* K3 surface is a pair (*X*, *j*), where *X* is a K3 surface and $j :\Lambda \hookrightarrow Pic (X)$ is a primitive embedding such that j(Λ) contains an ample line bundle.

From now on, let $\Lambda _{g,k}$ be the lattice of rank two with intersection matrix

2 $$\begin{aligned} \begin{bmatrix} 2g-2 & 2k \\ 2k & 0 \end{bmatrix}, \end{aligned}$$

where g>1 and k>0. Then by Dolgachev (1995) the moduli space $M_{\Lambda _{g,k}}$ of (ample) $\Lambda _{g,k}$-polarized K3 surfaces is a quasi-projective variety of dimension 18.

The following result generalizes the existence result of integral nodal rational curves on a generic polarized K3 surface $(X,L) \in \mathcal {F}_g$ to a generic $\Lambda _{g,k}$-polarized K3 surface.

#### Theorem 2.7

(Chen et al. 2022, Thm. 3.1) Let Λ be a lattice of rank two with intersection matrix as in (2) and suppose $L \in \Lambda$ is big and nef on a generic K3 surface $X \in M_{\Lambda }$. Then there exists an open dense subset $U \subset M_{\Lambda }$, such that for any X∈U the linear system |*L*| contains an integral nodal rational curve.

We now apply the above theorem to show that the curve of elliptic nodes $C_{en}$ is nonempty for a generic hyperelliptic K3 surface of genus g⩾3.

#### Corollary 2.8

Let n=0 or 1, r>n and g=2r+1-n. Then for a generic hyperelliptic K3 surface $(X,L_r)$ in the 18-dimensional locus $\mathcal {F}_{n,r} \subset \mathcal {F}_{g}$ (see Proposition 2.4), the curve of elliptic nodes $C_{en}$ of $(X,L_{r})$ is nonempty.

#### Proof

Let $(X,L_r) \in \mathcal {F}_{n,r}$ be a hyperelliptic K3 surface of genus g=2r-n+1. By construction, *X* admits a double cover $\pi :X \rightarrow \mathbb {F}_{n}$, and the polarization is given by $L_{r} = \pi ^{*}\mathcal {O}(e+rf)$. Define $M :=\pi ^{*}(f)$. The sublattice $\left\langle L_r, M \right\rangle \subset Pic (X)$ is isomorphic to $\Lambda _{g,1}$, so $(X,L_r)$ is $\Lambda _{g,1}$-polarized. We now apply Theorem 2.7 for the moduli space $M_{\Lambda _{g,1}}$. Since $\dim \mathcal {F}_{n,r} = \dim M_{\Lambda _{g,1}} = 18$, we deduce that for a generic $(X,L_r) \in \mathcal {F}_{n,r}$, the linear system $|L_r|$ contains an integral nodal rational curve. Finally, a deformation argument for nodal curves on K3 surfaces (Mori and Mukai 1983) shows that smoothening a node of such a rational curve yields a one-dimensional family of nodal elliptic curves in $|L_r|$. Therefore, the curve of elliptic nodes $C_{en}$ is nonempty for a generic hyperelliptic K3 surface in $\mathcal {F}_{n,r}$. □

### Proof of Theorem 0.2

We show below that for a generic K3 surface in the 18-dimensional hyperelliptic locus of $\mathcal {F}_g$ (see Corollary 2.8), the curve of elliptic nodes $C_{en}$ is a constant cycle curve. This immediately implies our main result Theorem 0.2: Conjecture 0.1 is true for an 18-dimensional family in each moduli space $\mathcal {F}_g$ with g⩾3.

#### Proposition 2.9

Let $(X,L_r)$ be a generic polarized K3 surface of genus g=2r-n+1 in $\mathcal {F}_{n,r}$, where $n \in \{0,1\}$ and r>n. Let $\pi :X \rightarrow \mathbb {F}_n$ be the double cover structure. Then the curve of elliptic nodes $C_{en}$ of $(X,L_r)$ is exactly the ramification locus of π. In particular, $C_{en}$ is a smooth constant cycle curve of genus 9.

#### Proof

By Corollary 2.8, the curve of elliptic nodes $C_{en}$ of a generic $(X,L_{r})$ in $\mathcal {F}_{n,r}$ is nonempty. Now we show that $C_{en} \subset X$ is a constant cycle curve. The strategy is similar to the double plane case (see Sect. 1.2). By construction, the hyperelliptic K3 surface *X* is a double cover $\pi :X \rightarrow \mathbb {F}_n$ branched over a smooth irreducible curve $B \in |-2K_{\mathbb {F}_n}|$. The pullback $\pi ^*:|e+rf| \rightarrow |L_{r}|$ of linear systems induces a bijection between the following two sets:

(i) irreducible rational curves in |e+rf| that are simply tangent to *B* at (g-1) points and intersect *B* transversally at the remaining intersection points,
(ii) nodal elliptic curves in $|L_{r}|$.

Moreover, the (g-1) points of tangency of such a rational curve *l* with *B* are pulled back to the (g-1) nodal points of $\pi ^{*}l$. Therefore, all nodal points of nodal elliptic curves in |*L*| belong to the ramification locus $\pi ^{-1}(B)$. By Definition 1.3, the curve of elliptic nodes $C_{en}$ of $(X,L_r)$ is exactly the ramification curve $\pi ^{-1}(B)$, which is smooth, irreducible and of genus 9. Finally, since $\mathbb {F}_{n}$ is rational, the natural involution on *X* is non-symplectic (see Sect. 1). From Proposition 1.2 it then follows that the fixed curve $\pi ^{-1}(B) = C_{en}$ is a constant cycle curve. □

#### Remark 2.10

*(Alternative Proof)* In the above setting, $C_{en}$ can be shown to be a constant cycle curve without using its description as the fixed locus of a non-symplectic involution.

Let $p :X \xrightarrow {\pi } \mathbb {F}_n \rightarrow \mathbb {P}^1$ be the natural elliptic fibration. Let $E \in |L_r| = |\pi ^{*}(e+rf)|$ be a nodal elliptic curve, and *P* be one of its nodal points. Denote by *F* be the fibre of *p* containing *P*. Since the fibre class $\pi ^{*}(f)$ satisfies $(\pi ^{*}(e+rf).\pi ^{*}(f)) = 2$, the fibre *F* intersects *E* precisely at the nodal point *P* with multiplicity two, giving [E].[F]=2[P] in $CH _0(X)$. By the property of the Beauville–Voisin class (see Sect. 1) we have $[E].[F] = 2c_X$, so $2[P] = 2c_X$. The torsion-freeness of $CH _0(X)$ then implies $[P] = c_X$. As this holds for every nodal point P∈E of any nodal elliptic curve $E \in |L_r|$, a generic point $P \in C_{en}$ satisfies $[P] = c_X$ in $CH _0(X)$ by Definition 1.3. It then follows from Voisin (2003, Lem. 10.7) that $C_{en}$ is a constant cycle curve, thereby recovering our main result.

## Further discussions on the conjecture

Throughout this section, let (*X*, *L*) be a polarized K3 surface of genus g>2 with nonempty Severi variety $V_{L,g-1}$. We introduce two conditions on nodal elliptic curves in |*L*| that ensure the curve of elliptic nodes $C_{en}$ is a constant cycle curve. The first condition is a necessary and sufficient criterion that applies to all pairs (*X*, *L*), while the second condition is stronger and only applies to hyperelliptic K3 surfaces, thus cannot be used to study Conjecture 0.1 for generic polarized K3 surfaces.

Let *E* be a nodal elliptic curve in |*L*|. Denote its (g-1) nodal points by $P_1, \ldots , P_{g-1}$ and write $\{P_{i}^{\prime },P_{i}^{\prime \prime }\}$ for the preimage of $P_i$ under the normalization map $\nu :\tilde{E} \rightarrow E$. Consider the following two conditions related to the curve *E*:

- $[P_i] = [P_j]$ in $CH _0(X)$ for any i≠j.
- $[P_{i}^{\prime }]+[P_{i}^{\prime \prime }] = [P_{j}^{\prime }]+[P_{j}^{\prime \prime }]$ in $CH _0(\tilde{E})$ for any i≠j.

### Lemma 3.1

In the above setting, we have the following:

(i) For every nodal elliptic curve E∈|L|, (b) implies (a).
(i) $C_{en}$ is a constant cycle curve if and only if (a) holds for every nodal elliptic curve E∈|L|.

### Proof

We first prove (i). Assume that (b) holds for a nodal elliptic curve E∈|L|. Pushing forward the equalities along the morphism $f :\tilde{E} \mathop {\twoheadrightarrow }\limits ^{\nu } E \hookrightarrow X$ gives $2[P_i] = 2[P_j]$ in $CH _0(X)$ for any i≠j. Since $CH _0(X)$ is torsion free (Rojtman 1980), it follows that $[P_i] = [P_j]$ in $CH _0(X)$ for any two nodal points $P_i,P_j$ of *E*. Therefore, (a) holds.

For (ii), the “only if” direction is immediate from the construction of $C_{en}$, so it suffices to prove the “if” direction. Let E∈|L| be a nodal elliptic curve. The double point formula (Fulton 1998, Thm. 9.3) applied to the morphism $f :\tilde{E} \rightarrow X$ gives $\sum _{i=1}^{g-1}[P_i^{\prime }] + [P_i^{\prime \prime }] = f^{*}[E]$ in $CH _0(\tilde{E})$. Applying the pushforward $f_{*}$ and the projection formula yields

$$ 2\sum _{i=1}^{g-1}[P_i] = f_{*}f^{*}[E] = [E]^{2} = (2\,g-2)c_X \in CH _0(X),$$

where the last equality is a property of the Beauville–Voisin class $c_X \in CH _0(X)$ (see Sect. 1). If (a) holds for *E*, then $[P_i] = c_X$ in $CH _0(X)$ (using the torsion-freeness of $CH _0(X)$). Hence if (a) holds for any nodal elliptic curve E∈|L|, then there is a dense open subset $U \subset C_{en}$ such that $[P] = c_X$ for every P∈U. From Voisin (2003, Lem. 10.7) it follows that $C_{en}$ is a constant cycle curve. □

The previous lemma provides a sufficient condition for $C_{en}$ to be a constant cycle curve, namely, that (b) holds for every nodal elliptic curve E∈|L|. However, as the following result shows, this condition holds precisely when (*X*, *L*) is a hyperelliptic K3 surface. Since hyperelliptic K3 surfaces form only a divisor in the moduli space, this condition cannot be used to study Conjecture 0.1 for generic polarized K3 surfaces.

### Proposition 3.2

Let (*X*, *L*) be a polarized K3 surface of genus g>2 with nonempty Severi variety $V_{L,g-1}$. Then (b) holds for all nodal elliptic curves in |*L*| if and only if (*X*, *L*) is a hyperelliptic K3 surface.

### Proof

We first prove the “if” direction. Suppose (*X*, *L*) is hyperelliptic, and let E∈|L| be a nodal elliptic curve. By Remark 2.10, for any nodal point *P* of *E*, there exists a fibre *F* of the natural elliptic fibration $p :X \rightarrow \mathbb {F}_n \rightarrow \mathbb {P}^1$ such that $[E].[F] = 2[P] \in CH _0(X)$. Let $P^{\prime }, P^{\prime \prime }$ be the preimages of *P* under the normalization $\nu :\tilde{E} \rightarrow E$, and let $f :\tilde{E} \xrightarrow {\nu } E \subset X$ be the composition. Then the class $[P^{\prime }]+ [P^{\prime \prime }] = f^{*}[F]$ in $CH _0(\tilde{E})$ is independent of the choice of the nodal point P∈E. Since E∈|L| was arbitrary, this shows that (b) holds for every nodal elliptic curve in |*L*|.

Now assume that (b) holds for all nodal elliptic curves in |*L*|. We show that (*X*, *L*) is hyperelliptic. By Saint-Donat (1974, Thm. 3.1), the existence of an irreducible curve in |*L*| ensures that the linear system |*L*| is base point free, hence it induces a morphism $\phi _{L} :X \rightarrow \mathbb {P}^g$. Fix a nodal elliptic curve E∈|L|. Denote its *i*-th node by $P_i$, and let $\{P_{i}^{\prime }, P_{i}^{\prime \prime }\}$ be the preimages of $P_i$ under the normalization $\nu :\tilde{E} \rightarrow E$.

The restriction $L|_{E}$ of the line bundle *L* to *E* also has no base points, and the induced morphism $\phi _{L|_{E}}$ is the restriction of $\phi _{L}$ to *E*. Consider the line bundle $M_i :=\mathcal {O}_{\tilde{E}}(P_i^{\prime }+P_{i}^{\prime \prime })$. Since $\tilde{E}$ is a smooth elliptic curve, the linear system $|M_i|$ is a $g_2^1$ and induces a degree-two morphism $\phi _{M_{i}} :\tilde{E} \rightarrow \mathbb {P}^1$. By construction, the morphism $\phi _{M_{i}}$ factors through *E*. Let $\nu _{g-1} :\mathbb {P}^1 \rightarrow \mathbb {P}^{g-1}$ be the Veronese embedding. Then we obtain the following diagram where the right square commutes:

**Figure:**
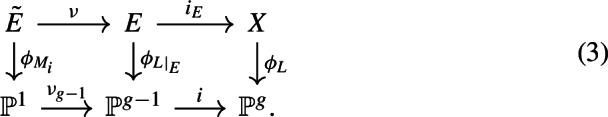

We claim that the outer rectangle of (3) also commutes (up to an automorphism of $\mathbb {P}^{g}$) and that this is enough to imply the statement. Indeed, assuming the claim, every nodal elliptic curve E∈|L| maps generically 2:1 onto its image under $\phi _L$. Since the one-dimensional family of nodal elliptic curves in |*L*| dominates *X*, it follows from Theorem 2.1 that $\phi _L$ is a double cover onto its image. Hence by definition (*X*, *L*) is a hyperelliptic K3 surface.

Now we prove the claim. It suffices to show that the following two pullbacks of the line bundle $\mathcal {O}_{\mathbb {P}^g}(1)$ are isomorphic:

$$\begin{aligned}&(i \circ \nu _{g-1} \circ \phi _{M_{i}})^{*}\mathcal {O}_{\mathbb {P}^g}(1) \simeq \phi _{M_{i}}^{*}(\mathcal {O}_{\mathbb {P}^1}(g-1)) \simeq \mathcal {O}_{\tilde{E}}((g-1)(P_i^{\prime }+P_{i}^{\prime \prime })) \\ &(\phi _{L} \circ i_{E} \circ \nu )^{*}\mathcal {O}_{\mathbb {P}^g}(1) \simeq (i_{E} \circ \nu )^{*} \mathcal {O}_X(E) \simeq \mathcal {O}_{\tilde{E}}\left( \sum _{i=1}^{g-1}(P_i^{\prime } + P_{i}^{\prime \prime })\right) . \end{aligned}$$

The last isomorphism follows from the double point formula (Fulton 1998, Thm. 9.3) applied to the morphism $i_E \circ \nu :\tilde{E} \rightarrow X$. By assumption, we have $[P_i^{\prime }] + [P_i^{\prime \prime }] = [P_j^{\prime }] + [P_{j}^{\prime \prime }]$ in $CH _0(\tilde{E}) \simeq Pic (\tilde{E})$ for all *i*, *j*, hence the two pullbacks above are isomorphic. This completes the proof. □

## Data Availability

Data sharing is not applicable to this article as no datasets were generated or analysed in this work.

## Footnotes

1 In Huybrechts (2014), the above notion is called a *pointwise* constant cycle curve, while the original definition of a *constant cycle curve* requires the class $[\eta _C] - c_X \times k(C) \in CH ^2(X \times k(C))$ associated to the generic point $\eta _C \in C$ to be torsion. These two notions are proved by Huybrechts to be equivalent over uncountable algebraically closed fields, hence in our case we do not distinguish them.
